# The Acute Metabolic Impacts of Kampferia parviflora Extract in Healthy Men: A Randomized, Double-Blind, Proof-of-Concept Study

**DOI:** 10.7759/cureus.81561

**Published:** 2025-04-01

**Authors:** Cassandra Evans, Douglas Kalman, Lia Jiannine, Tony Ricci, Peter Byers, Flavia Pereira, Viraaj Miriyala, Jose Antonio

**Affiliations:** 1 College of Health Care Sciences, Nova Southeastern University, Davie, USA; 2 Nutrition, Nova Southeastern University Dr. Kiran C. Patel College of Osteopathic Medicine, Davie, USA; 3 Psychology and Neuroscience, Nova Southeastern University, Davie, USA; 4 Exercise and Sport Science, Nova Southeastern University, Davie, USA; 5 Exercise Science, Keiser University, West Palm Beach, USA; 6 College of Osteopathic Medicine, Nova Southeastern University, Davie, USA

**Keywords:** ginger extract, lipid metabolism, nutrition and metabolism, respiratory exchange ratio, resting metabolic rate

## Abstract

Background

Obesity and its related comorbidities are a major health concern, with numbers increasing globally. There is a need for innovative approaches to prevent obesity or mitigate the negative health effects. Research suggests that ginger consumption has an anti-obesity effect through various mechanisms, including changes in lipid metabolism and increases in thermogenesis. This study assessed the effects of a ginger-containing supplement on energy expenditure and substrate utilization.

Methods

Ten males volunteered for this double-blind, two-dose crossover, proof-of-concept study. Resting energy expenditure (REE) and respiratory exchange ratio (RER) were assessed prior to supplementation and throughout both study visits. After consuming a ginger-containing supplement (Gyngerlean™ 100 or 200 mg doses), REE was assessed at 60, 120, and 180 minutes at each visit. There was a minimum 24-hour washout period between the two study visits.

Results

No significant differences were observed at baseline between the 100 mg and 200 mg doses for REE (100 mg: 2203 ± 497 kcal vs. 200 mg: 2454 ± 501 kcal; p = 0.2408) or the RER (100 mg: 0.79 ± 0.09 vs. 200 mg: 0.81 ± 0.04; p = 0.4911). Post-dosing, the 100 mg dose showed no significant changes in REE or RER over all time points. For the 200 mg dose, REE remained stable over all time points (no significant change), while the RER showed a significant reduction at 120 and 180 minutes post-consumption (p < 0.05).

Conclusion

This exploratory study demonstrated increased fat oxidation following acute ingestion of a ginger-containing supplement (200 mg), suggesting the potential role of ginger in weight and body composition management. Future studies are needed to further the understanding and potential application of this finding. More research is warranted.

## Introduction

The obesity epidemic affects 650 million people worldwide, and the rate continues to rise at an alarming pace [1-3]. Currently, 38% are overweight/obese globally and by 2030 the US is projected to have obesity rates reaching 78% [3]. Research shows that individuals with obesity have an increased risk of comorbidities such as cardiovascular disease, type 2 diabetes, dyslipidemia, and metabolic syndrome (MetS) [4]. Obesity develops when there is an imbalance between energy intake and expenditure, leading to increases in body mass and body fat. Approaches to address obesity and related comorbidities include lifestyle behavior modification, surgery, pharmacotherapy, and alternative therapies. Recently, functional foods have gained attention for their role in improving health [5,6]. There is evidence to suggest that functional foods, including traditional herbs, may support weight management and metabolic health [6].

One such herb is *Zingiber officinale*, commonly known as ginger or yellow ginger. It is a plant with a long history of use in cooking and herbal medicine. Extensive research has demonstrated the effects of ginger’s bioactive compounds, including antioxidant, antimicrobial, anti-neuroinflammation, anticancer, and alleviation of chemotherapy-induced nausea and vomiting [7-10]. Ginger has been found to reduce weight, waist-to-hip ratio, and fasting glucose levels and increase high-density lipoprotein (HDL) cholesterol, with no reported adverse effects [7,11,12]. Moreover, ginger’s bioactive compounds may aid in weight management. These bioactive compounds influence weight through different mechanisms, including appetite suppression, thermogenesis, regulation of lipid metabolism, and improved insulin sensitivity [7,13]. Additionally, different types of ginger contain different bioactive compounds, potentially offering distinct benefits.

*Kaempferia parviflora*, commonly referred to as Thai black ginger, is a plant species belonging to the *Zingiberaceae* family. Traditionally, black ginger has been used for its stomach-protective, allergy-relieving, and fatigue-reducing properties. Numerous animal studies have demonstrated the anti-obesity effects of *Kaempferia parviflora,* ranging from increased energy expenditure, lipolysis, a decrease in body mass, and alterations in lipid metabolism [14-17]. Matsushita et al. [18] examined the effects of acute *Kaempferia parviflora* ingestion on energy expenditure and its relationship to brown adipose tissue (BAT). After consuming a placebo or supplement (100 mg of *Kampferia parviflora* extract), resting energy expenditure (REE) was assessed at multiple time points (15, 45, and 75 minutes) using a respiratory gas analyzer. BAT was assessed using PET and CT scans after cold exposure, which involved placing participants' feet on ice blocks. Subjects were classified as high or low based on the observed BAT activity. Compared to the placebo, the supplement group’s energy expenditure significantly increased (229 ± 69 kJ/day) at 60 minutes. A greater (351 ± 50 kJ/day) increase in energy expenditure was observed in the high BAT group only. The authors suggest that *Kampferia parviflora* extract increases energy expenditure via BAT activation [18]. Yoshino et al. [19] examined the effects of chronic *Kaempferia parviflora* on abdominal body fat in older, overweight adults. Following 12 weeks of supplementation (150 mg *Kampferia parviflora* extract), visceral fat, subcutaneous fat, and total fat significantly decreased compared to the placebo group. Additionally, the supplement group experienced a greater decrease in triglycerides and fasting blood glucose, further supporting the anti-obesity effects of *Kampferia parviflora* extract.

Another herb of interest is *Alpinia galangal* or red ginger. It is prominent in Southeast Asia and commonly used as a flavoring in foods. This herb has a history of use in traditional medicine due to its bioactive phenolic compounds, including galangoisoflavonoid and methyleugenol [20]. Sivakumar et al. [21] examined the effects of galangin, a flavonoid present in *Alpinia galangal*, on whole-body insulin resistance and kidney oxidative stress in rodents. Rodents were fed a high-fructose diet to mimic MetS. The experimental groups received galangin extract (50, 100, and 200 μg/kg body weight, respectively) for 60 days. In a dose-dependent manner, galangin normalized blood glucose and insulin levels and prevented changes in kidney function. This study demonstrates the *Alpinia galangal* extract's ability to positively influence insulin sensitivity and reduce renal damage in rodents with diet-induced MetS. Similarly, Kumar and Alagawadi [22] reported anti-obesity effects in mice supplemented with galangin while consuming a high-fat diet. The authors suggest that several mechanisms are responsible for these results. Pancreatic lipase activity was reduced in the supplement group. The findings imply that the ingredient alters lipid digestion and decreases absorption. The supplement group consumed significantly less food than the control group, suggesting an anorexigenic effect. The supplement group had reductions in triglycerides, total cholesterol, and liver weight. This is most likely related to the changes in lipid metabolism.

There is sufficient evidence to support the further exploration of ginger in weight management and metabolic health. However, little is known about the benefits of combining different types of ginger. Thus, the purpose of this investigation was to assess the potential effects of two different doses of a study product, GyngerLean™, on REE and respiratory exchange ratio (RER), which contains *Zingiber officinale* (yellow ginger), *Alpinia galangal* (red ginger), and *Kaempferia parviflora* (black ginger). It is hypothesized that GyngerLean™ may impact energy expenditure and fat oxidation, and this exploratory randomized double-blind clinical study sought to obtain objective data to aid in the overall understanding of how ginger may impact aspects of metabolism.

## Materials and methods

Study design

This study followed a randomized, double-blinded, crossover design. The participants reported to the clinical site on two separate visits. The participants were instructed to abstain from exercise on the testing day and arrive at the lab fasted (no food or caffeine ingestion for 4 hours prior). Testing sessions consisted of two visits per week with a minimum of 24 hours between visits. During the first visit, demographics and anthropometrics were assessed. At every testing, each subject completed the following protocol: baseline metabolic rate and respiratory quotient (RQ). Immediately after the baseline test, the participants consumed supplement 1 or 2. Metabolic rate and RQ testing were repeated at 60, 120, and 180 minutes after supplementation. All participants underwent an informed consent process by the Declaration of Helsinki and an approved IRB protocol approved by the institutional review board at Nova Southeastern University, with IRB number 2024-319.

Participants

All participants were healthy adult men. The participants were excluded from the study if they were unhealthy, had a body mass index below 25 or above 30 (i.e., 21 or 35), were taking prescribed medication or over-the-counter medication known to affect the metabolic rate, or had a history of mental health/psychiatric treatment in the 12 months prior to the study. The participants were instructed to maintain a stable lifestyle with no change in exercise or diet for the duration of the study, which was assessed via self-report. See Table 1.

**Table 1:** Baseline characteristics All data are presented as the mean and standard deviation. A sample size of 10 males was chosen for this study.

Characteristic	Average
Age (years)	26.9±5.6
Height (cm)	179.6±9.3
Body mass (kg)	89.0±11.7
Body mass index	27.8±1.8
Lean body mass (kg)	73.5±14.1
Fat mass (kg)	15.5±6.0
% Body fat	18.0±6.4
Total body water (liters)	53.9±10.5

Metabolic and RQ testing

An indirect calorimetry device (Parvo Medics TrueOne 2400; Parvo Medics, Salt Lake City, UT) was used to measure resting metabolic rate (RMR) and RQ. The device was calibrated according to the manufacturer’s instructions. The participants were instructed to lie completely still and relax for 15 minutes. The first 5 minutes were allotted for acclimatization. The Parvo Metabolic Cart measures the volume of oxygen consumed (VO_2_) and the volume of carbon dioxide produced (VCO_2_) by the patient on a breath-by-breath basis. The ratio of these gases is used to determine the RQ, providing information regarding substrate utilization. The parameters that were measured and used for analysis were RMR, O_2_ volume (VO_2_), CO_2_ volume (VCO_2_), the RQ, percentage of utilized fat (Fat%), and percentage of utilized carbohydrates (CHO%).

Intervention

The participants were randomly assigned to consume supplement 1 (GyngerLean™ 100 mg) or supplement 2 (GyngerLean™ 200 mg) on the first visit. The order was reversed for the second visit. GyngerLean™ is composed of *Alpinia galangal* (red ginger), *Kaempferia parviflora* (black ginger), and *Zingiber officinale* (yellow ginger). The dietary supplement was provided by Cepham Inc. (Somerset, NJ). Both study products were identical in size, shape, and color.

All data are expressed as mean ± SD. Basic descriptive statistics were used for participant demographics and anthropometrics. A paired t-test was used to assess any differences between the 100 and 200 mg doses for REE and RER at baseline. To determine the effects of Gyngerlean™ on energy expenditure, a repeated-measures analysis of variance (ANOVA) was used to determine differences between 100 mg and 200 mg doses for the 60-, 120-, and 180-minute time intervals. A repeated Dunnett's multiple comparisons test was used as the post hoc statistical method to compare the means of the 1-, 2-, and 3-hour time points against the baseline (i.e., control). Dunnett's test compares each treatment group only to the control, which results in a more robust test (i.e., a lower chance of type II errors or false negatives) since fewer comparisons are made. A p-value of <0.05 was considered significant.

## Results

**Figure 1:**
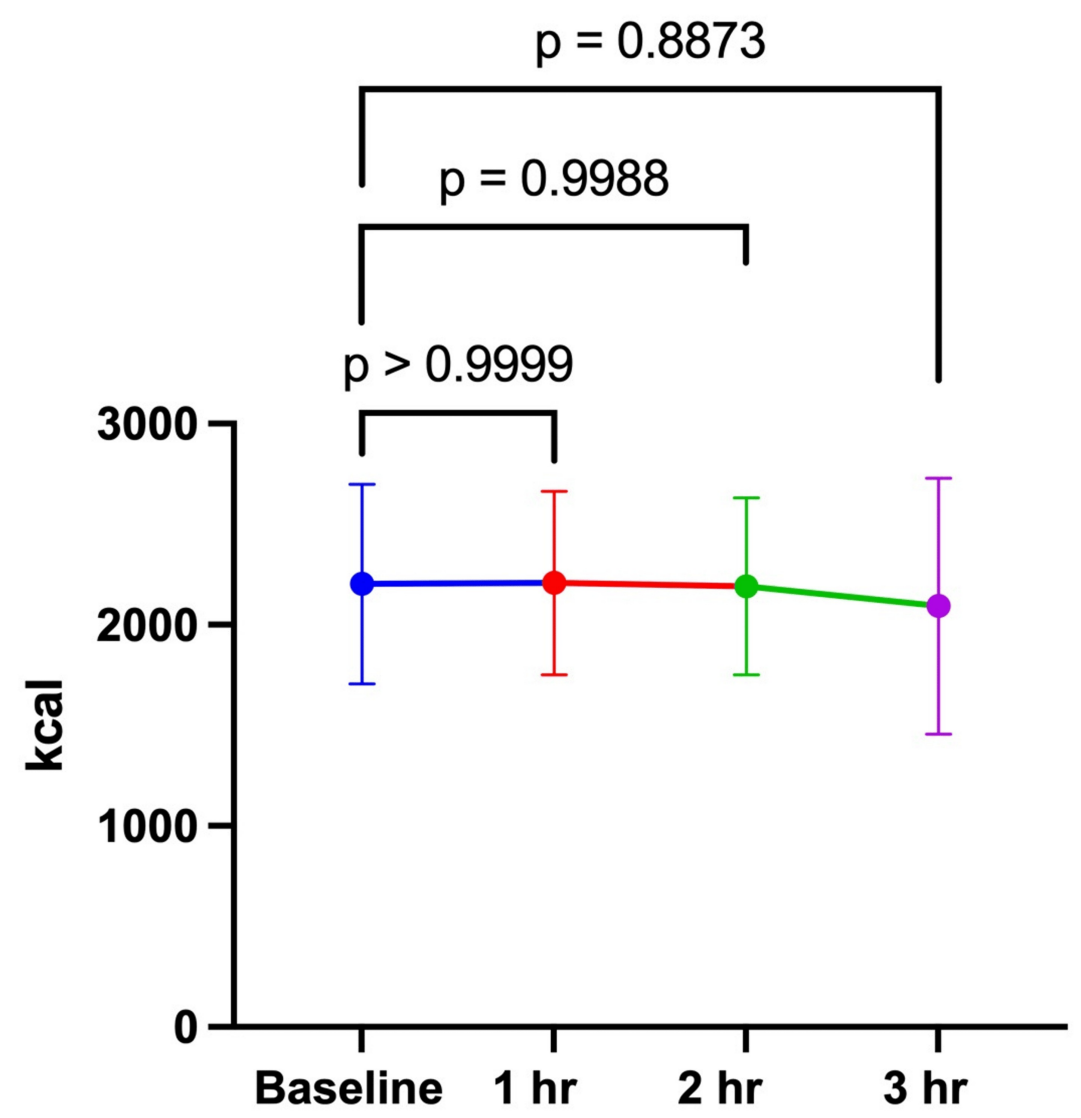
REE 100 mg REE over 3 hours after consuming 100 mg of the investigational product. The data in the figure are expressed as the mean and standard deviation. REE, resting energy expenditure.

**Figure 2:**
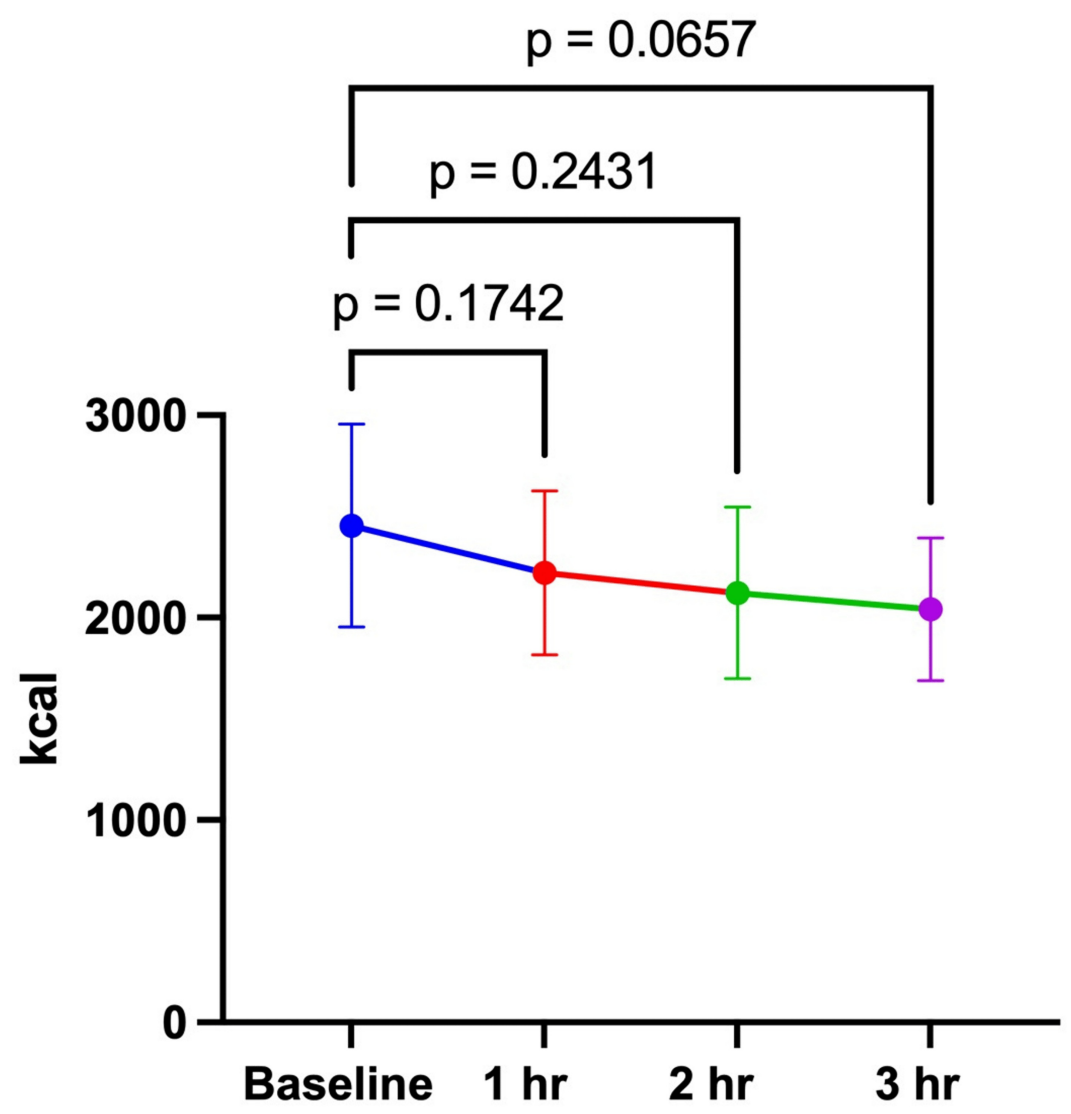
REE 200 mg REE over 3 hours after consuming 200 mg of the investigational product. The data in the figure are expressed as the mean and standard deviation. REE, resting energy expenditure.

**Figure 3:**
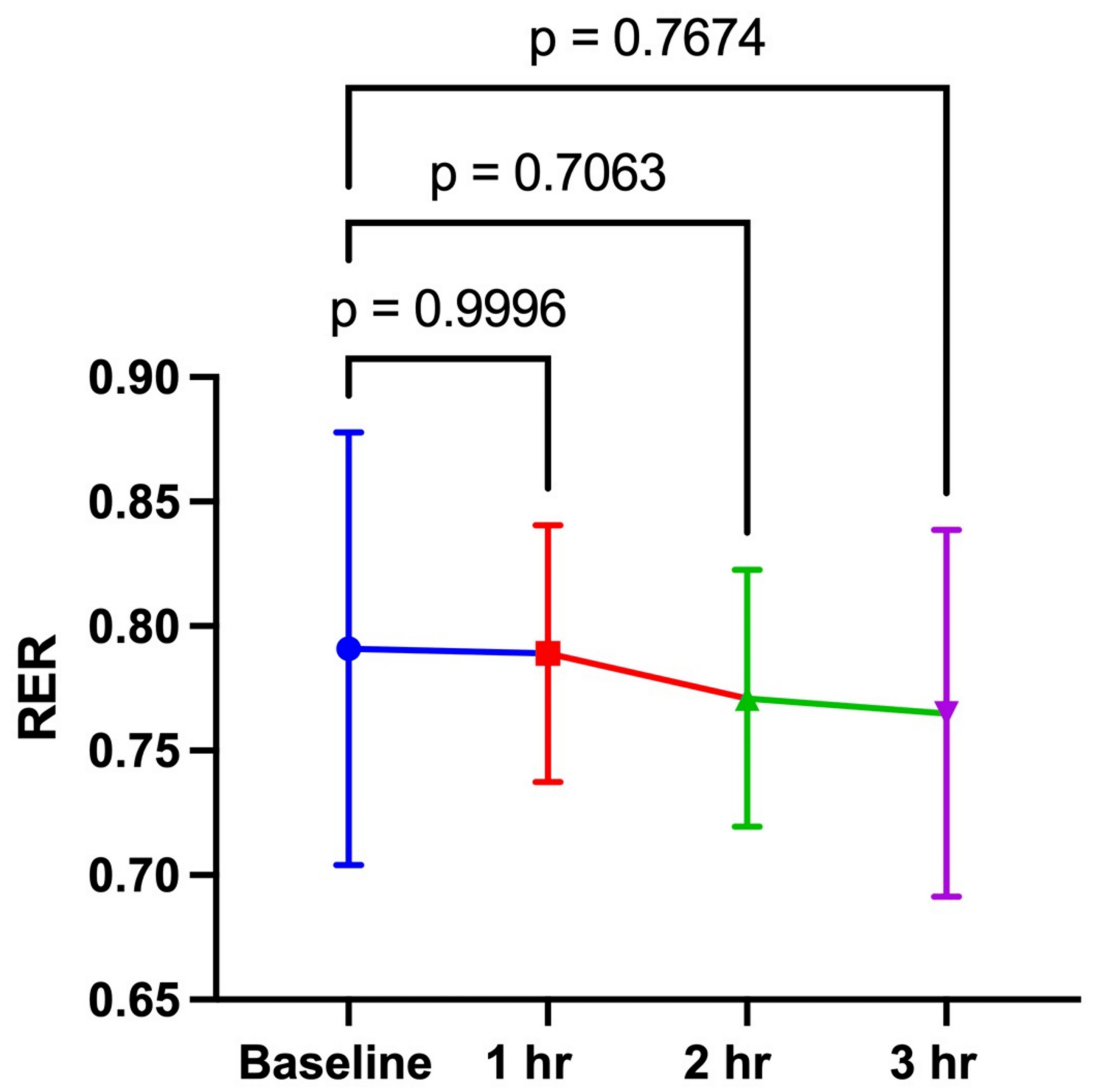
RER 100 mg RER over 3 hours after consuming 100 mg of the investigational product. The data in the figure are expressed as the mean and standard deviation. RER, respiratory exchange ratio.

**Figure 4:**
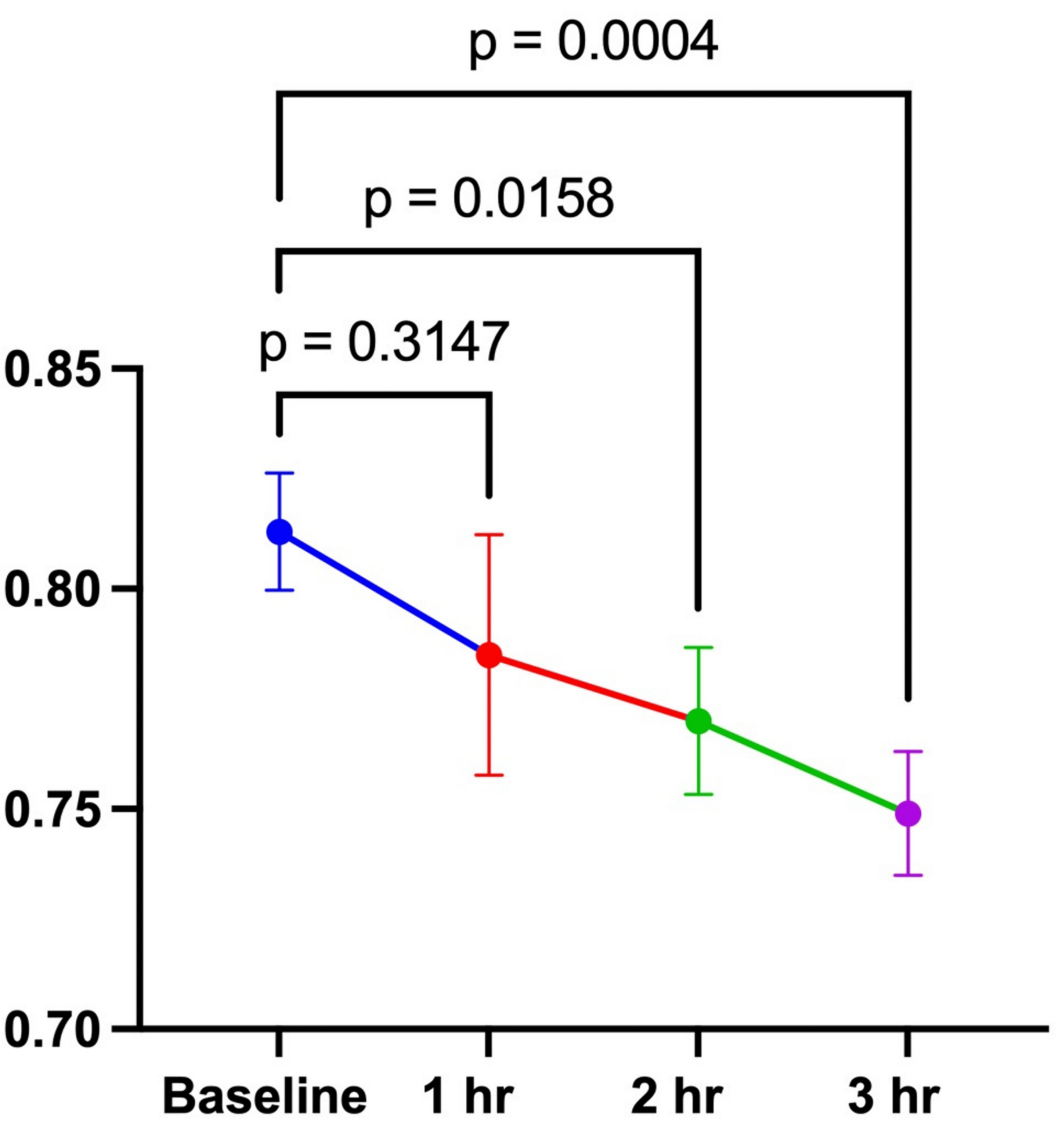
RER 200 mg RER over 3 hours after consuming 200 mg of the investigational product. The data in the figure are expressed as the mean and standard deviation. There were no significant differences between the baseline assessment and 1-hour post-consumption assessment. However, there were significant differences between the baseline assessment and 2- and 3-hour post-consumption assessments. RER, respiratory exchange ratio.

The study was conducted from July 23, 2024, to August 8, 2024. Ten male research participants completed both arms of the investigation (Table 1). There were no significant differences between the 100 and 200 mg doses at baseline for REE (100 mg: 2203 ± 497 kcal vs. 200 mg: 2454 ± 501 kcal; p = 0.2408) or RER (100 mg: 0.79 ± 0.09 vs. 200 mg: 0.81 ± 0.04; p = 0.4911). For the 100 mg dose, there were no significant differences in REE or RER between the baseline assessment and 1-, 2-, and 3 hours post-consumption of the investigational product (Table 2, Figures 1, 2). For the 200 mg dose, there were no significant differences over time for REE between baseline and all timepoints (Table 3, Figure 3). RER was significantly lower at 2 (0.77 ± 0.05) and 3 hours (0.75 ± 0.04) post-consumption (Figure 4). This study was registered with Clinical Trials (NCT06805201).

**Table 2:** RER over 3 hours after consuming 100 mg and 200 mg of the investigational product. RER, respiratory exchange ratio. *p = 0.0158; **p = 0.0004.

	100 mg			200 mg		
	Mean ± SD	Std. Error	p-Value	Mean ± SD	Std. Error	p-Value
Baseline	0.7910 ± 0.08685	0.02747		0.8130 ± 0.04191	0.01325	
1 hour	0.7890 ± 0.05152	0.01629	0.9996	0.7850 ± 0.08618	0.02725	0.3147
2 hours	0.7710 ± 0.05152	0.01629	0.7063	0.7700 ± 0.05292*	0.01673	0.0158
3 hours	0.7650 ± 0.07367	0.02330	0.7674	0.7490 ± 0.04433**	0.01402	0.0004

**Table 3:** REE over 3 hours after consuming 100 mg and 200 mg of the investigational product. REE, resting energy expenditure.

	100 mg		200 mg	
	Mean ± SD	Std. Error	Mean ± SD	Std. Error
Baseline	2203 ± 496.7	157.1	2454 ± 501.4	158.5
1 hour	2208 ± 456.0	144.2	2222 ± 405.7	128.3
2 hours	2192 ± 440.1	139.2	2122 ± 424.5	134.2
3 hours	2093 ± 636.7	201.3	2042 ± 352.5	111.5

## Discussion

The present study investigated the effects of two different doses (100 mg and 200 mg) of an investigational product (Gyngerlean™) on REE and RER in healthy males (n = 10). 

Our findings indicate that both doses (100 and 200 mg) of the investigational product did not produce any significant changes in REE. These findings suggest that a larger dose and/or chronic consumption may be needed to increase REE. Similar to our findings, Fagundes et al. [23] and Mansour et al. [13] reported no changes in the energy expenditure following consumption of a ginger-containing supplement. In both studies, the participants consumed the supplement or placebo, followed by breakfast. Energy expenditure was assessed via respiratory gas exchange at multiple time points. However, a true comparison is difficult to make due to the energy expenditure being assessed postprandially. Moreover, Fagundes et al. [23] recruited healthy weight females, while the participants in Mansour et al.'s [13] study were obese based on BMI. Contrary to the results of the current study, others have reported increases in energy expenditure. Matsushita et al. [18] reported a significant increase in energy expenditure following acute consumption of *Kaempferia parviflora* extract. A greater increase in energy expenditure was reported when participants were subdivided based on the levels of BAT. The participants were young males with a healthy weight BMI (21.2 ± 0.3). These differences in findings may be due to the preparation and amount of the ginger extracts used.

Our findings show that the 100 mg dose did not affect RER; however, the 200 mg dose did elicit a change. RER was significantly lower at 2 and 3 hours post-consumption. Changes in RER show a shift in substrate utilization. As time progressed after consuming 200 mg of the investigational product, a greater reliance on fat oxidation relative to carbohydrate oxidation was observed. Previous studies suggest that ginger influences fat oxidation through different mechanisms. Seo et al. [24] reported increased expression of fibroblast growth factor 21 (FGF21), acyl-CoA oxidase 1 (ACOX1), and carnitine palmitoyltransferase 1 (CPT1). Li et al. [25] reported an inhibitory effect of ginger in mice on cyl CoA carboxylase (Acc) expression and phosphoenolpyruvate carboxy kinase 1 (Pepck1). These studies suggest that ginger may influence fat oxidation by altering gene expression related to fat oxidation and lipogenesis. Mansour et al. [13] reported an increase in the thermic effect of food after consuming a ginger-containing supplement with food. Both human and animal models suggest that ginger supplementation activates BAT, thereby increasing thermogenesis [17,18,26]. The exact mechanisms of action regarding the effect of Gyngerlean™ on substrate utilization are presently unclear. However, the research suggests that ginger may alter fat oxidation via altered gene expression, and increase lipolysis and thermogenesis. This study was a proof of concept and, as such, has certain limitations in design. The within-subject design used in this study presents a limitation, as there may be changes in RER when at rest. Future studies should utilize acute and chronic doses in appropriate populations with a larger sample size and different study designs to determine if effects are still observed. 

## Conclusions

Within the confines of this study, the 100 mg and 200 mg ginger products did not impart a significant change in REE in the post-consumption period. However, the 200 mg ginger product dosage demonstrated a significant impact on the RER during 2 and 3 hours post-ingestion, suggesting an enhanced fat oxidation effect of the product. Future studies are needed to better understand the ginger product (Gyngerlean™) and its impact on metabolism and substrate utilization. Future research should address whether chronic use would impact weight and body composition status.
